# The influence of the size and aspect ratio of anisotropic, porous CaCO_3_ particles on their uptake by cells

**DOI:** 10.1186/s12951-015-0111-7

**Published:** 2015-09-04

**Authors:** Bogdan Parakhonskiy, Mikhail V Zyuzin, Alexey Yashchenok, Susana Carregal-Romero, Joanna Rejman, Helmuth Möhwald, Wolfgang J Parak, Andre G Skirtach

**Affiliations:** Shubnikov Institute of Crystallography, Russian Academy of Science, Moscow, Russia; Institute of Nanostructures and Biosystems, Saratov State University, Saratov, Russia; Fachbereich Physik, Philipps University of Marburg, Marburg, Germany; Department of Interfaces, Max-Planck Institute of Colloids and Interfaces, Potsdam, Germany; NanoBio-Photonics, Ghent University, Ghent, Belgium; Department of Molecular Biotechnology, Ghent University, Ghent, Belgium

**Keywords:** Calcium carbonate, Anisotropic, Uptake, Cells, Internalisation

## Abstract

**Background:**

Recent reports highlighting the role of particle geometry have suggested that anisotropy can affect the rate and the pathway of particle uptake by cells. Therefore, we investigate the internalization by cells of porous calcium carbonate particles with different shapes and anisotropies.

**Results:**

We report here on a new method of the synthesis of polyelectrolyte coated calcium carbonate particles whose geometry was controlled by varying the mixing speed and time, pH value of the reaction solution, and ratio of the interacting salts used for particle formation. Uptake of spherical, cuboidal, ellipsoidal (with two different sizes) polyelectrolyte coated calcium carbonate particles was studied in cervical carcinoma cells. Quantitative data were obtained from the analysis of confocal laser scanning microscopy images.

**Conclusions:**

Our results indicate that the number of internalized calcium carbonate particles depends on the aspect ratio of the particle, whereby elongated particles (higher aspect ratio) are internalized with a higher frequency than more spherical particles (lower aspect ratio). The total volume of internalized particles scales with the volume of the individual particles, in case equal amount of particles were added per cell.

**Electronic supplementary material:**

The online version of this article (doi:10.1186/s12951-015-0111-7) contains supplementary material, which is available to authorized users.

## Background

A multitude of physicochemical properties of particles such as size [1, 2], shape [3–7], charge [8, 9], surface chemistry [10–15], the aggregation state and colloidal stability [16], as well as stiffness [17, 18] directly influences their uptake, intracellular trafficking, and even exocytosis [19] and cytoskeleton re-organization [20]. Different cell types have been used in the experiments including macrophages [21, 22]. Furthermore, these properties are also known to affect particles’ in vivo circulation, retention, and bio-distribution [23–26]. Because these diverse parameters are highly entangled [27], experimental determination of the effect of just one parameter remains challenging [28]. For example, the aggregation state plays a particularly important role as it may outweigh the influence of size and charge. On the other hand, the formation of the protein corona simultaneously affects both the aggregation state of particles and their uptake [29]. Therefore, a meaningful analysis necessitates that particles appear in the non-aggregated state in the medium in which they are applied to cells. In case of non-agglomerated particles, the shape of particles can also play a substantial role in their interaction with cells [30, 31]. While the particle shape alone will most likely not determine the pathway by which particles are taken up by cells [32], there are studies indicating that the shape can significantly affect the kinetics of internalization [33]; although in some reports different results have been published [34]. The above conclusions apply to both micrometer and nanometer sized particles.

Several studies were conducted to characterize internalization of non-spherical particles and to compare it to spherical particles. For example, polystyrene particles of various sizes and shapes were used to study their internalization by alveolar macrophages [35]. It was found that particles of all shapes were taken up by phagocytosis. However, the internalization velocity [36] depended on the angle between the particle and the initial contact point with the macrophage membrane before internalization. This angle determined whether macrophages initiate uptake or simply spread on the particles [35]. The influence of particle orientation on the interaction between particles and the plasma membrane has been reported by Gilbert et al. [37]. The geometry of the polymer particles was found to influence the rate of endocytosis between disk-shaped particles and spheres of the same volume. Non-spherical particles were reported to have a lower uptake than spherical particles [38] attributed to a larger curvature radius. Furthermore, disk-shaped “flat” particles (i.e. oblate spheroid shape with a low aspect ratio) were reported to have slower internalization velocity in the first hours of incubation with endothelial cells, but the accumulation of particles around the nucleus was similar to that in the long term [33]. This is consistent with the study of Shimoni et al. who reported that the uptake of “flat” polyelectrolyte capsules with decreasing aspect ratio (i.e. ratio of height to width) was lower than the uptake of spherical capsules with the same composition [5]. In that study, the fate of the capsules was also analyzed, and capsules were reported to be localized in lysosomal compartments regardless of their shape. In an additional report, Rubner et al. [39] claim that uptake of flat particles is slowed down to an extent that the particles reside as “backpacks” on the cellular plasma membrane. In a different trend, it was reported that HeLa cells internalize non-spherical hydrogel particles via several different mechanisms, and that they internalize elongated, rod-like particles (i.e. prolate spheroid shape, high aspect ratio) at a higher rate [40]. Although this trend does not hold for lengthy fibers (i.e. for very large aspect ratios); they were found to be internalized at a lower rate than that for round particles [41]. Furthermore, Kolhar et al. [42] reported similar nonspecific uptake for spherical and rod shaped particles in brain and lung endothelial cells using static cell cultures, microfluidics and in vivo studies in mice. These results were further substantiated by a mathematical model. The above mentioned studies were conducted under somewhat different conditions, which may lead to contradictory results. Data from a number of different studies may lead to the general perception that flat particles (i.e. oblate spheroid shape, low aspect ratio) are internalized by cells less efficiently than spherical ones (i.e. aspect ratio = 1), which, in turn, are internalized at a slower rate than particles elongated in one dimension (i.e. prolate spheroid shape, high aspect ratio) as was presented by Chithrani et al. [1]. However, most of these studies suffer from the fact that in addition to the shape of particles typically also the size, aggregation state, and surface chemistry influence their uptake. Furthermore, the results depend on the nature of the particles and type of cells. For example, a recent study made a claim opposite to that outlined above: that flat (i.e. two-dimensionally elongated) as well as rod-like shaped (i.e. one-dimensionally elongated) particles are internalized faster than spherical ones [43]. This only accentuates the need for further studies to create a more comprehensive picture. We note that anisotropy of particles can be achieved by synthesizing them with a higher aspect ratio (“intrinsic anisotropy”) or simply by controlling their patchiness (“extrinsic anisotropy”) [44–46]. Anisotropic particles adsorb and orient themselves differently even at the border of two different media [47].

In our present work we fabricated anisotropic, polyelectrolyte-coated porous calcium carbonate microparticles (CaCO_3_) of different shapes and different aspect ratios. These particles were used to investigate the impact of shape on the particle internalization by cervical carcinoma cells. Porous CaCO_3_ microparticles [48, 49] are attractive because of their acceptable biocompatibility, high surface area to volume ratio, high loading capacity for cargo molecules [50], and low synthesis costs. These particles serve the function of versatile templates for the assembly of hollow polyelectrolyte microcapsules [51–55]. The capsules, fabricated on various templates [56], may be either spontaneously internalized by cells [32, 57–59], or specifically targeted to cells [60]. The porosity of CaCO_3_, which is often used as a template or core for capsules [48, 56], is one of the key features regarding their application in drug delivery [61], and it has been employed for entrapping dyes, polymers, proteins, polypeptides, inorganic nanoparticles, etc. into the inner volume for sensor applications [62], enzyme-catalyzed reactions [63, 64] or intracellular uptake of capsules [65–68] by cells. In a commonly used preparation method of such particles equal amounts of salt concentrations are mixed producing spherical particles.

In this work we have prepared three different shapes: porous spherical, ellipsoidal, and cuboidal particles [69], using a new method in which unequal concentrations of the interacting salts were used, and compare them with porous spherical calcium carbonate as well as commercially available smooth silica particles serving as control. The particles were coated with polyelectrolytes, so that the surface charges are adjusted, while on the other hand this coating could allow constructing capsules with the possibility of controlled release [70–74]. During the course of our work, we have also varied the sizes [75] of calcium carbonate particles using yet another innovative approach, in which ethylene glycol was used to slow down the course of the reaction, thus providing data on the influence of the size of particles on their uptake by cells.

## Results and discussion

**Fig. 1:**
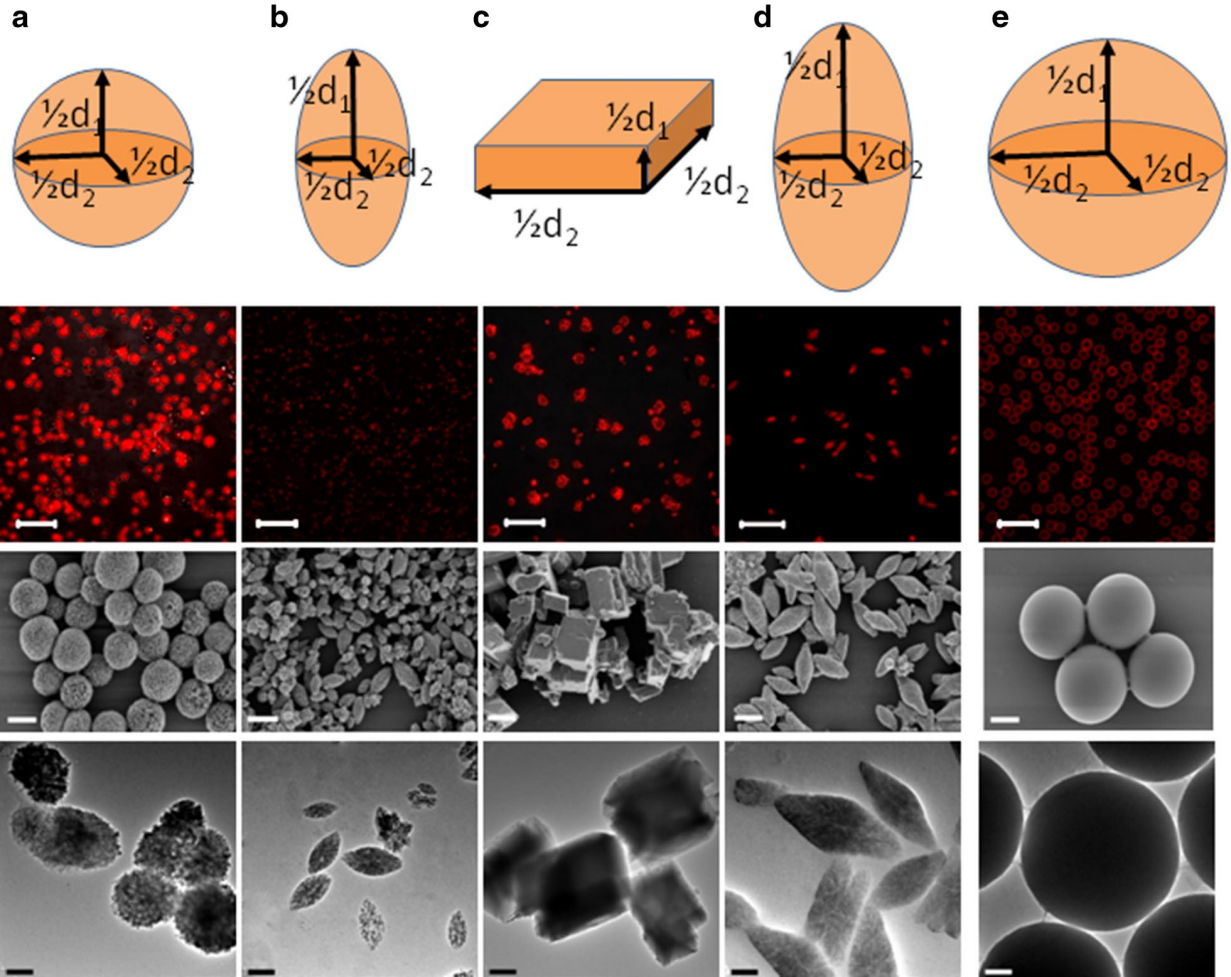
The *first row* from the *top*: schematics of the geometry of the investigated particles. **a** “Small” spherical CaCO_3_ particles, **b** “small” ellipsoidal CaCO_3_ particles, **c** cuboidal CaCO_3_ particles, **d** “big” ellipsoidal CaCO_3_ particles, and **e** control “big” spherical SiO_2_ particles. All particles have symmetry in the x–y plane. *d*_*1*_ describes the extension of the particles along the axis of symmetry, and *d*_*2*_ the extension perpendicular to this axis. The *second row* from the *top*: fluorescence microscopy images of the different particles used in the uptake study as recorded in solution for (**a**) through (**e**). The *scale bars* in all images correspond to 20 µm. The *third row* from the *top*: SEM images of the different particles used in the uptake study for (**a**) through (**e**). The *scale bars* in all images correspond to 2 μm. The *fourth row* from the *top*: TEM images of the different particles used in the uptake study for (**a**) through (**e**). The *scale bars* in all images correspond to 1 μm.

**Fig. 2:**
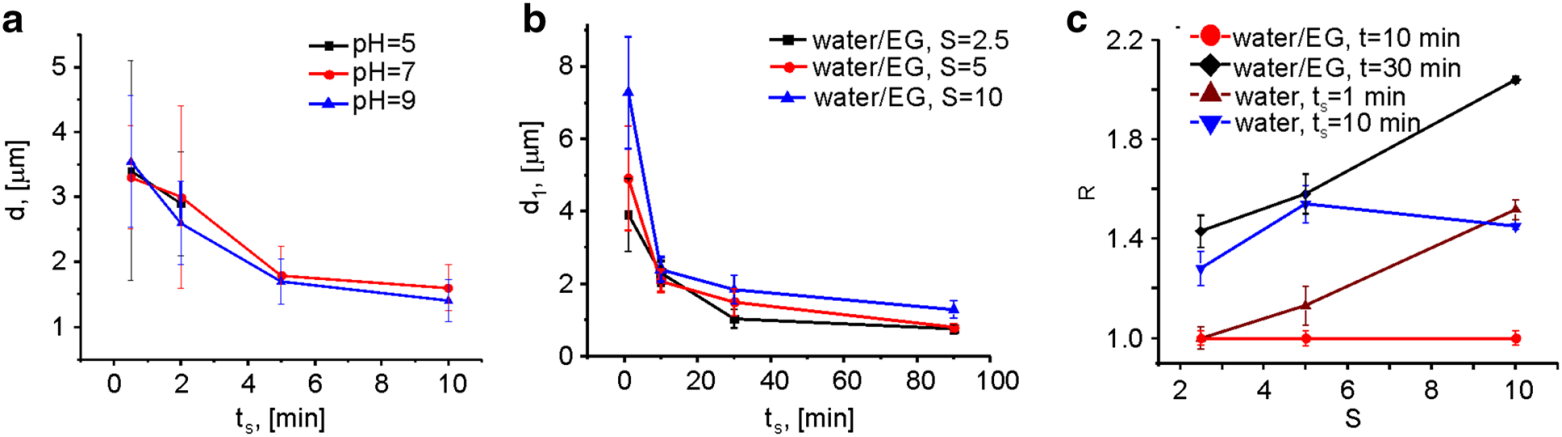
Tuning the sizes of particles. **a** The size d = d_1_ = d_2_ of spherical CaCO_3_ particles was tuned by varying the stirring time t of the CaCl_2_ and Na_2_CO_3_ solutions at pH values of 5, 7, 9 in pure water solution with a sodium carbonate to calcium chloride ratio S = 1. **b** The size of the long axis d_1_ of ellipsoidal CaCO_3_ particles was tuned by varying the stirring time t_s_ at certain salt concentration ratios S = 2.5 (*black curve*); 5 (*red curve*); and 10 (*blue curve*) in water/ethylene glycol = 1/5 solutions of pH = 9.5. **c** The aspect ratio R (d_1_/d_2_) of the CaCO_3_ particles as a function of S; R was tuned by varying the stirring time t_s_ in different water and water/EG (ethylene glycol) mixtures at pH = 9.5. The *vertical bars* represent the standard deviation values.

In order to obtain calcium carbonate particles of different shapes, *cf.* Fig. 1, several parameters were adjusted during their synthesis: the stirring time *t*_*s*_, the presence/absence of ethylene glycol in the aqueous solution in which CaCl_2_ and Na_2_CO_3_ were mixed, the sodium carbonate to calcium chloride ratio S, and the pH of the CaCl_2_ and Na_2_CO_3_ solutions. By adjusting the stirring time (and to a little extent also the pH of the CaCl_2_ and Na_2_CO_3_ solutions), while keeping the concentrations of CaCl_2_ and Na_2_CO_3_ equal, we were able to fabricate spherical particles of different sizes (Fig. 2a). The solubility of CaCO_3_ particles increases by decreasing the pH [76, 77]. At pH = 5 the solubility of CaCO_3_ particles reaches a critical point, and, if they are stirred at this pH for longer than 2 min, they completely dissolve. Thus, at low pH the size of CaCO_3_ particles cannot be modified by controlling the stirring time for longer than 2 min, *cf.* Fig. 2a. Contrary, at higher pH values the size can be better controlled by altering the stirring time. Figure 2a shows the influence of the stirring time obtained with different salt solution pHs (5, 7, 9) when mixing 1 mL of each Na_2_CO_3_ and CaCl_2_ (both 0.33 M, i.e. S = 1). The size of the particles decreased when the stirring time was increased, in agreement with the results reported by Sawada et al. [78]. The decrease of particle size was explained by these authors by assuming a phase transition from the unstable vaterite to the stable calcite. This transformation is due to the higher solubility of vaterite compared to calcite. Therefore, the phase transition is energetically favorable [78]. Increasing the pH value from 7 to 9 further decreases the particle size to only a little extent, *cf.* Fig. 2a.

Anisotropic growth could be also achieved by varying the composition of the solvent, e.g. by using mixtures of ethyleneglycol (EG) and water. The presence of EG in the reaction solution decreases the calcium carbonate solubility [75] and changes the time of the reaction between CaCl_2_ and Na_2_CO_3_ from minutes to hours. We found that, after the first 30 s of stirring, the lengths of the longest axis of the particles d_1_ were around 3 and 6 μm, at salt ratios S = 2.5 and S = 10, respectively (Fig. 2b). Extending the mixing time to 10 min resulted in the formation of particles with d_1_ ≈ 2 μm regardless of salt concentration ratio. The size of the particles slightly decreased to approximately d_1_ ≈ 1 μm upon further stirring. The shape, however, can be also modified in the absence of EG by increasing the salt concentration ratio and the stirring time. Figure 2c shows the change of aspect ratio R = d_1_/d_2_ at different salt concentrations S, two different stirring times t_s_, and presence/absence of EG. Overall this approach enables the control of the CaCO_3_ particle size with d_1_ between 0.5 and 6 μm with aspect ratios ranging from 1 to 2. The synthesis of cuboidally shaped particles requires extended time, i.e. stirring, for at least 3 h, in water. Table 1 gives a summary of the obtained CaCO_3_ particle geometries and of the commercial SiO_2_ particles used for the cellular uptake study. We note that although a direct comparison between smooth silica and porous calcium carbonate particles is difficult, the influence of the porosity could be assessed. Inherent monodispersity of silica particles would be an additional factor in this assessment.

**Table 1:** Particles with different shapes and composed of different materials were used to study their uptake by HeLa cells

Material	Shape	d_1_ (μm)	d_2_ (μm)	V (μm^3^)	R
CaCO_3_	“Small” spherical	3.3 ± 0.8	3.3 ± 0.8	18.8	1
CaCO_3_	“Small” ellipsoidal	1.7 ± 0.2	0.9 ± 0.2	0.7	2.0
CaCO_3_	Cuboidal	2.5 ± 0.9	3.2 ± 1.1	25.6	0.8
CaCO_3_	“Big” ellipsoidal	3.1 ± 0.5	1.7 ± 0.8	4.7	1.8
SiO_2_	“Big” spherical	4.8 ± 0.2	4.8 ± 0.2	58.0	1

The length of the particle extension along the highest symmetry axis d_1_ (polar axis), and the width d_2_ (equatorial axis) of the particle extension in the plane perpendicular to the highest symmetry axis are given, *cf.* Fig. 1. From d_1_ and d_2_ the particle volume (V) and aspect ratio (R) were calculated. Data represent the mean value and standard deviation of 30–50 analyzed particles.

**Fig. 3:**
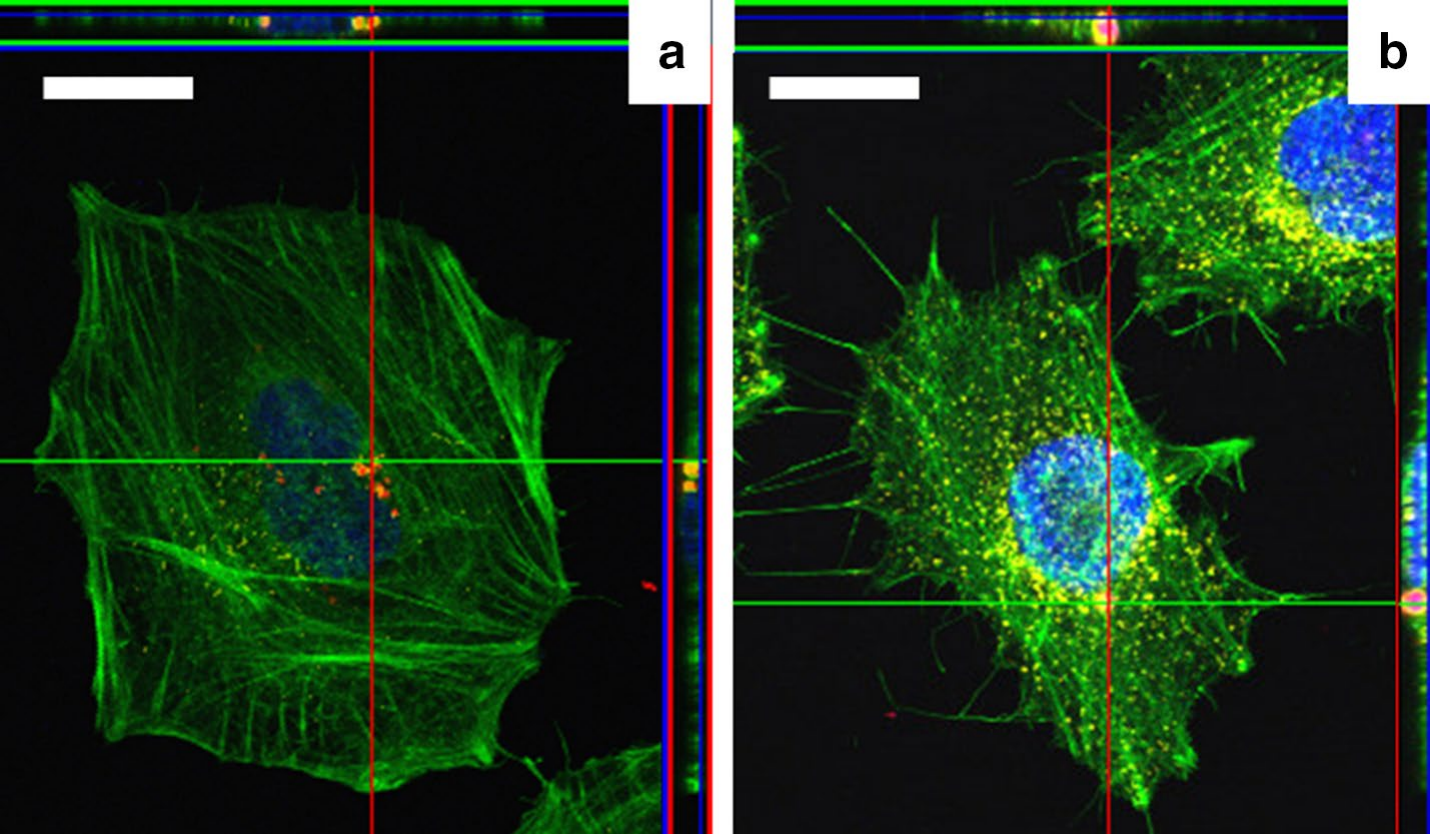
Orthogonal view from different planes (*x*/*y*, *x*/*z* or *y*/*z*) of the confocal microscope images used to analyze the particle uptake. Examples correspond to: **a** “small” ellipsoidal CaCO_3_ particles and **b** “big” spherical SiO_2_ particles (used as control). Co-localization of fluorescently labeled CaCO_3_ particles (with TRITC, in *red*) with the lysosomal marker Anti-LAMP1 labeled with DyLight 649 (artificially colored in *yellow*). The HeLa cell´s nucleus was stained with DAPI (in *blue*) and the cytoskeleton with Oregon Green^®^ 488 phalloidin (in *green*). Scale bars correspond to 20 µm.

**Fig. 4:**
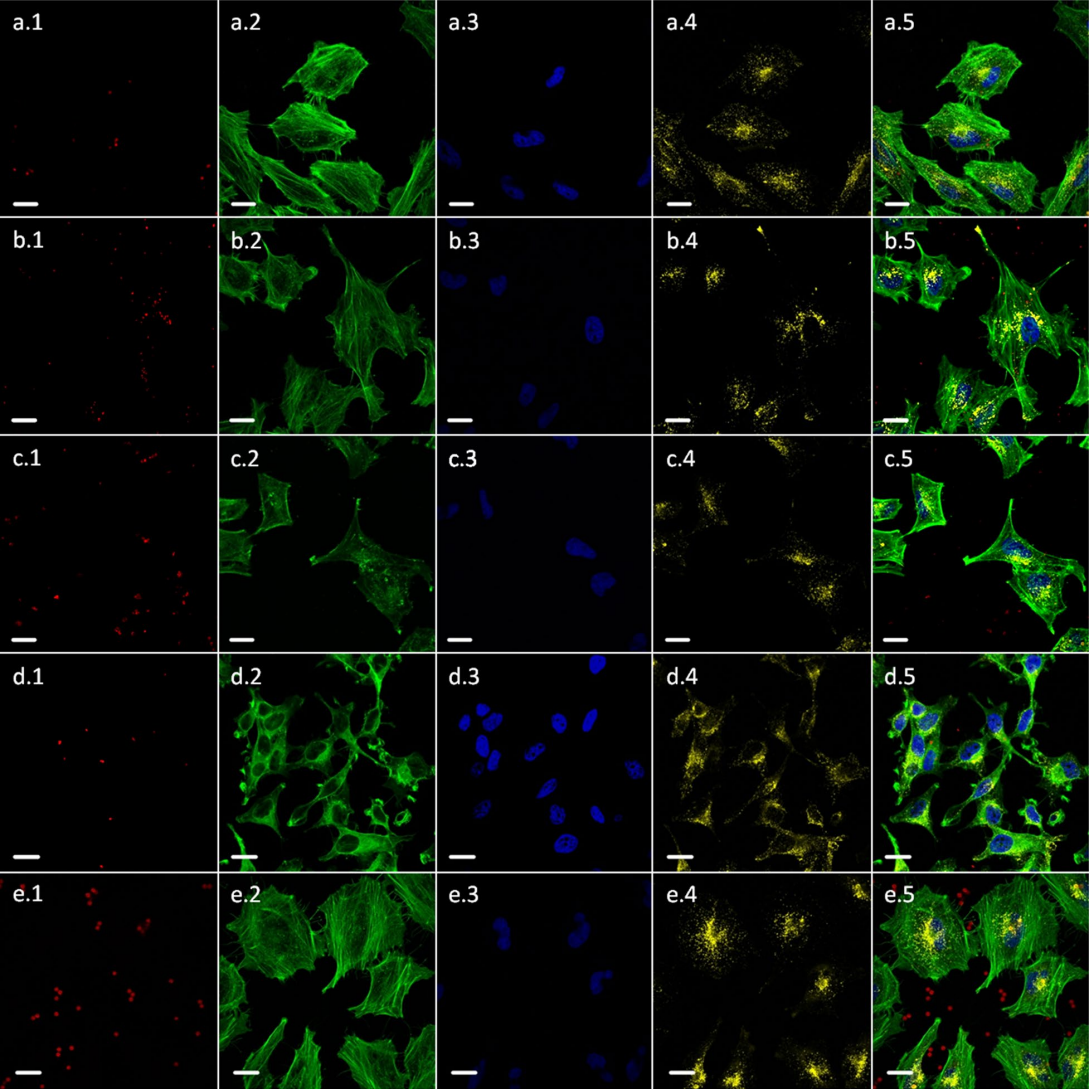
Confocal images of HeLa cells which internalized differently shaped particles (*red fluorescence*) after incubation for 24 h. **a** “Small” spherical CaCO_3_ particles, **b** “small” ellipsoidal CaCO_3_ particles, **c** cuboidal CaCO_3_ particles, **d** “big” ellipsoidal CaCO_3_ particles, and **e** “big” spherical SiO_2_ particles (used as control). Particles are labeled with TRITC (*red*). Nuclei, lysosomes, and cytoskeletons are fluorescence labeled with DAPI (*blue*), Anti-LAMP1 labeled with DyLight 649 (artificially colored in *yellow*), and Oregon Green^®^ 488 phalloidin (*green*), respectively. *1*, *2*, *3*, *4* and *5* mean the *red*, *green*, *blue* and *yellow* channels and the merged image, respectively. The *scale bars* in all images correspond to 20 μm.

**Fig. 5:**
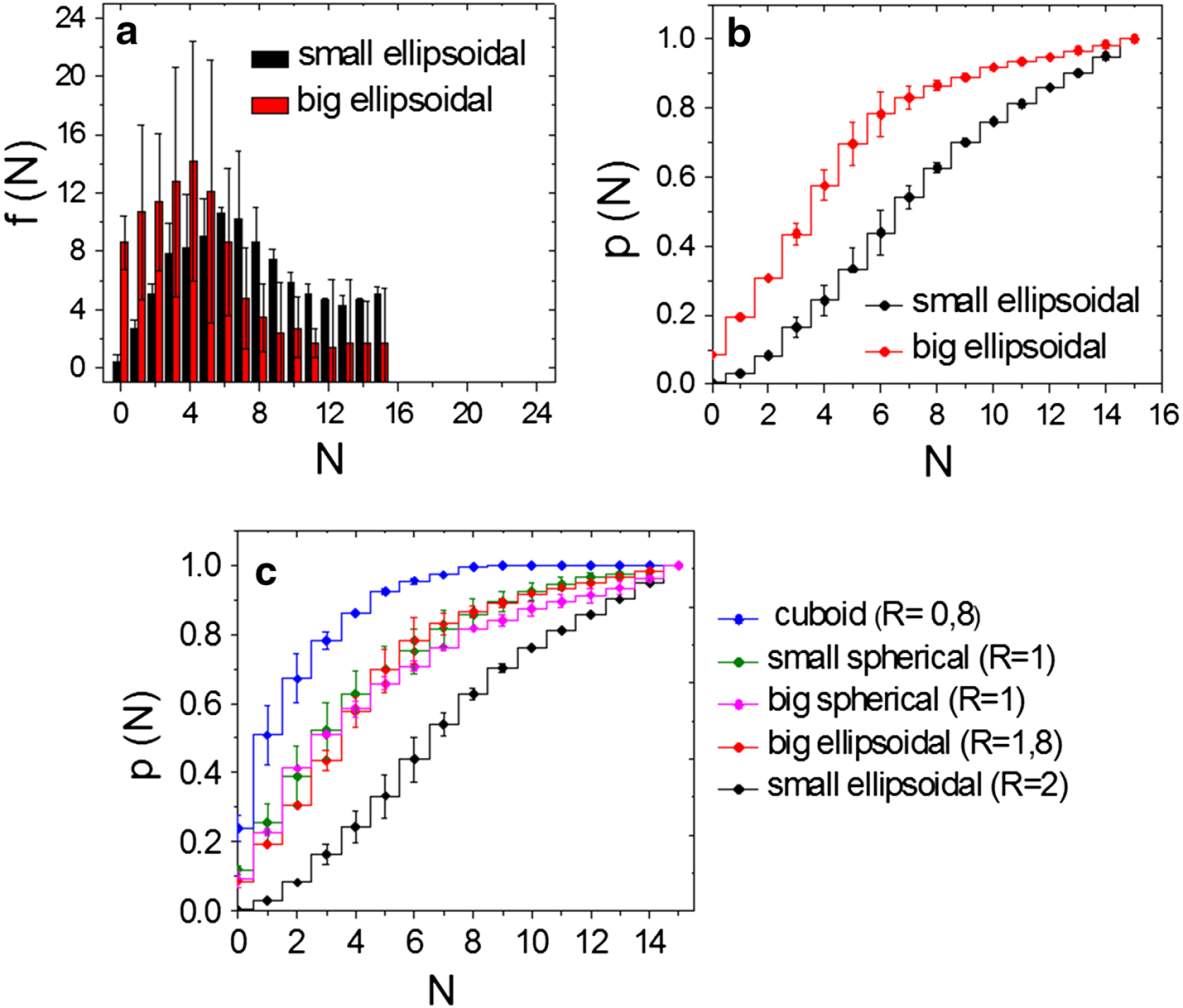
Influence of size on the uptake of ellipsoidal particles by HeLa cells. **a** Histogram of the frequency f(N) of cells which have internalized N particles per HeLa cell after 24 h of particle incubation with a concentration of 10 added particles per cell. **b** Corresponding cumulative probability plot p(N) showing a higher internalization rate for smaller ellipsoidal CaCO_3_ particles. The *vertical bars* represent the standard deviation values. **c** Cumulative probability p(N) for N internalized particles per cell for particles with different shape (and size) after 24 h of particle incubation with a cocentration of 10 added particles per cell. Cuboidal CaCO_3_ (*black dots*), “small” spherical CaCO_3_ (*green dots*), “big” spherical SiO_2_ (*pink dots*), “big” ellipsoidal CaCO_3_ (*red dots*), and “small” ellipsoidal (*black dots*). All particles had the same surface modification (positively charged layer of PAH). The *vertical bars* represent the standard deviation values.

The number of internalized particles per cell was evaluated after 24 h incubation by counting the particles inside HeLa cells via fluorescence microscopy and subsequent image analysis. A 24-h-incubation period was chosen because different endocytic processes proceed with different kinetics (e.g. clathrin-mediated endocytosis proceeds faster than uptake via caveolae). Therefore, a long incubation period ensured that the only parameter influencing the efficiency of uptake was the particle shape and size. The cell cytoskeleton, the nucleus and lysosomes were fluorescently stained and the co-localization of the red fluorescently labeled microparticles within the cellular compartments was evaluated with z-stack images as shown in Fig. 3. Figure 4 shows fluorescence images of HeLa cells that had internalized the differently shaped particles, which are located in the lysosomes, in agreement with previous work [32]. Note that multiple endocytotic pathways are responsible for particle internalization [32]. The histograms shown in Fig. 5a and b represent the number of cells f(N) which internalized the number of N particles and the corresponding cumulative distribution functions (CDFs) p(N) for two sizes of ellipsoidal particles. This figure is an example to show how the CDFs were calculated for all the samples. In particular, smaller ellipsoidal particles were internalized in higher number compared to bigger particles of similar shape. In Fig. 5c the CDFs of “small” spherical, cuboidal, “small” and “big” ellipsoidal and control “big” spherical” particles are shown, which have been obtained similarly to the description of Fig. 5a and b. Cuboidal particles were internalized to the lowest degree. Note that these cuboidal particles had a similar volume as the spherical CaCO_3_ particles. Thus differences in their internalization indicate the influence of the particle shape (assuming these are individually dispersed particles). Calcium carbonate “small” spherical and “big” ellipsoidal particles, as “big” spherical silica particles were taken up by the cells to similar extent, even though the silica particles have a much larger volume than the CaCO_3_ particles (58.0 µm^3^ for “big” spherical SiO_2_ compared with 18.8 and 4.7 µm^3^ for “small” spherical and “big” ellipsoidal CaCO_3_, respectively, *cf.* Table 1). “Small” ellipsoidal particles were internalized to a much higher extent than all other particles, pointing at a correlation between the aspect ratio of particles and their uptake. The higher the aspect ratio the better particles are internalized, which is independent of the microparticle size in first order approximation, *cf.* Fig. 5c.

While confirming the influence of particle shape on cellular uptake, it is necessary to critically discuss the significance of our data. The objective significance is limited by several issues. Firstly, as it can be seen from Fig. 1 there is some variation in shape (and size) of the particles within each batch. Standard particles, such as silica spheres used in this study as control are characterized by a very narrow size distribution. The differently shaped CaCO_3_ particles as prepared in this study were less mono-disperse. Secondly, it is challenging to vary only one parameter, such as shape, while keeping other parameters, such as size, constant. In the present study the main parameter to be varied was the particle shape, yet as presented in Table 1 it was accompanied by some variations in particle size. Introduction of a scaling parameter, the aspect ratio, helped to demonstrate shape-dependent particle uptake, which in first order was not affected by particle size. Thirdly, the physico-chemical properties of the particles may alter significantly once they are dispersed in cell media (containing proteins and salt). One of the most critical parameters in this context is the stability against agglomeration [8, 13]. Obviously shape- and size-related effects will be altered if particles are no longer individually dispersed, but agglomerated [28]. In this study, particles of the same surface chemistry, i.e. the same outer polyelectrolyte coating, were compared. Thus, while we certainly cannot exclude the presence of agglomerates, still the influence of the cell medium on the aggregation state of different particles should be comparable. Fourthly, in case of limited stability purification is restricted. In Fig. 1 (bottom row) for example, some traces of impurities, besides the actual particles, can be observed. Taken together these limitations indicate that statements have to be interpreted with care. However, our study is in line with several other ones wherein the complex influence of shape is pointed out [37], i.e. wherein the particle uptake is studied analyzing one single parameter—the aspect ratio. Our data confirm that flat particles (R < 1) are incorporated with a lower rate than elongated particles (R > 1). To be more precise, in case the same amount of particles is added to cells, each cell incorporates, on average, a higher number N of elongated in comparison with flat particles.

Quantification of particle uptake by cells in terms of the number of incorporated particles per cell may not always constitute the most appropriate metrics. For example, in the case of drug delivery the relevant measure is the amount of delivered payload or molecular cargo. This can be quantified by presenting the delivered volume per cell V_t_. The larger the volume V of a particle, the higher the amount of cargo delivered. The delivered volume per cell can be estimated by calculating the number N of particles internalized per cell multiplied by the volume V (*cf.* Table 1) of each particle (V_t_ = N·V). In this way the CDFs can be converted from number distribution p(N) CDFs into volume distribution p(V_t_), *cf.* Additional file 1: Figure S1. The volume distributions demonstrate, that in case cells are exposed to the same amount of particles (with different particle volumes V), the internalized total volume of particles depends on the volume of the individual particles, *cf.* Additional file 1: Figure S1. In other words, in case the same amount of “small” and “big” microparticles is added to cells, the total incorporated volume is higher for the bigger particles, in first order independent of the aspect ratio of the particles. Similar results were obtained when quantifying the delivered surface per cell (Additional file 1: Figures S1, S2). The internalized total surface of particles was higher for larger particles. This indicates that the metrics for quantifying particle uptake by cells is of utmost importance [26], and results will depend on the selection of the parameters which are investigated. Upon particle uptake (while keeping the number of added particles per cell constant) the number of internalized particles N scales with the aspect ratio of the particles, whereas the total volume of all internalized particles V_t_ depends on the particle volume V.

We note that development of anisotropic particles [79–84] and capsules [85–88] is seen to influence the transport through the membranes [89], which is significant and can be traced even with the ion level precision [90], and eventual uptake by cells. Such developments are of interest not only for intracellular delivery, but also for artificial and multicompartment capsules [91–93]. In the present work we only investigated one type of cells, one type of surface coating, and one time, and the anisotropy was varied. While the other parameters certainly influence particle–cell interaction as analyzed in previous studies both experimentally [9, 18, 32] and theoretically [36], focus of this work was on analyzing one homologous study, in which only shape (as related to the volume) of the particles was varied.

## Conclusions

We have prepared porous calcium carbonate particles with different geometries using a novel method controlling physico-chemical parameters such as pH of solutions, the ratio of salt concentrations, and the duration of the reaction. Subsequently, these particles were used for studying uptake by living cell. The data shown in our study point-out that cellular uptake of particles can be related to simple physico-chemical properties of the particles. One of the most important aspects in cellular uptake studies, often challenging to implement in practice, is to exclusively control the studied parameter. In the presented studies, two parameters are varied: the aspect ratio of the particles and their sizes, i.e. volumes; the influence of these parameters on cell uptake was analyzed.

Our results point out that the internalization rate is increased with increased aspect ratio (excluding very long fibers, wires). If equal amount of particles is added per cell, then the total volume of internalized particles scales with the volume of the individual particles. Smooth, monodisperse silica particles were used an additional control system. The used particle system, porous polyelectrolyte multilayer covered calcium carbonate particles, offers a number of advantages: inexpensiveness, possibility to vary the aspect ratio, porosity, as well as a possibility to control such physicochemical properties as surface charge and surface chemistry. These advantages make the current system an attractive drug delivery platform.

## Methods

### Materials and methods

#### For microparticle formation

Poly(allylamine hydrochloride) (PAH, M_w_ = 70 kDa), poly(sodium 4-styrenesulfonate) sodium salt (PSS, M_w_ = 70 kDa), tetramethylrhodamine isothiocynate-dextran (TRITC-dextran, M_w_ = 70 kDa), calcium chloride dihydrate (CaCl_2_·2H_2_O), and sodium carbonate (Na_2_CO_3_) were purchased from Sigma-Aldrich, Germany. Ethylene glycol solution (99%) was purchased from Alfa Aesar. In all procedures Milli-Q water was used with resistivity >18 MΩ.

#### For cell culture

Trypsin–EDTA was purchased from GIBCO. Dulbecco’s Essential Minimum Eagle’s Medium (EMEM), Penicillin–Streptomycin (P/S) was delivered by Sigma-Aldrich. Phosphate Buffered Saline (PBS) and fetal serum bovine were purchased from Biochrom.

#### For immunostaining

The primary monoclonal mouse anti-human LAMP1 antibody was obtained from the Developmental Studies Hybridoma Bank. The secondary antibody donkey anti-mouse IgG (H + L) conjugated with DyLight 649 and bovine serum albumin (BSA) *IgG*-*free* were delivered by Jackson Immuno Research Laboratories. (4′,6-diamidino-2-phenylindol, dilactate) (DAPI) and Oregon Green^®^ 488 phalloidin were purchased from Life Technologies. Saponin from quillaja bark (≥10%) was delivered by Sigma-Aldrich. Fluoromount-G was obtained from SouthernBiotech. Glycine (≥99%) was purchased from Roth. A confocal scanning microscope LSM 510 Meta (Zeiss), equipped with a diode laser (405 nm; 30 mW), an Ar-laser (458, 477, 488,514 nm; 30 mW), a HeNe-laser (543 nm; 1 mW), and a HeNe-laser (633 nm; 5 mW) from Carl Zeiss was used for the uptake studies. The staining protocols used for the uptake studies are explained in detail in the Additional file 1 and have been reported previously by Kast et al. [30].

### Synthesis of calcium carbonate particles

Calcium carbonate microparticles of various shapes, Fig. 1, were fabricated using a combination of unequal salt concentration and addition of ethylene glycol [69] while controlling the reaction mixing time and pH of the solutions. Briefly, for spherical CaCO_3_ microparticles 1 mL of Na_2_CO_3_ (0.33 M, pH 8) was injected into a glass vessel and then an equal volume of CaCl_2_ (0.33 M, pH 12) was injected and stirred at 600 rpm for 1 min. This time the pH was not adjusted. Further, the diameter of spherical particles was tuned from 3.5 to 1.5 µm by changing the pH of the salt solutions (CaCl_2_ and Na_2_CO_3_), by addition of HCl/NaOH, and the stirring time as it is described in Fig. 2a. Small ellipsoidal CaCO_3_ particles (1.7 ± 0.1 µm) were produced in a mixture of water and ethylene glycol. 1 mL of Na_2_CO_3_ (1 M, pH = 9.5) was injected into a glass vessel containing 10 mL ethylene glycol (EG) under stirring. Then 1 mL of CaCl_2_ (0.1 M, pH = 9.5) was mixed with 10 mL of EG; it was subsequently thoroughly stirred and was added to the glass vessel containing 1 mL of Na_2_CO_3_ and 10 mL of EG. The mixture was further stirred at 600 rpm for 30 min. The pH of the salt solutions was adjusted to 9.5 by addition of HCl/NaOH. The sodium carbonate to calcium chloride concentration ratio, S, was in this case equal to 10. The obtained particles had 1.7 ± 0.1 µm length for the longest axis (d_1_), 0.9 ± 0.2 µm for the shortest axis (d_2_) and an aspect ratio R = d_1_/d_2_ = 2. The size of ellipsoidal particles could be also tuned by changing the stirring time and by slightly modifying the salt concentration ratio, *cf.* Fig. 1. Large ellipsoidal CaCO_3_ particles were synthesized in water (without EG) at conditions identical to that of small ellipsoidal particles. Cuboidally shaped particles were prepared by mixing 1 mL CaCl_2_ (1 M, pH 8) and 1 mL Na_2_CO_3_ (1 M, pH 12) in a glass flask under stirring (without EG). The mixture was further stirred at 600 rpm for at least 3 h. All particle solutions were thoroughly washed immediately after the synthesis with water and alcohol in order to remove un-reacted components and residual ethylene glycol. Then the particles were placed for 1 h in a conventional oven set at 70°C. All differently shaped CaCO_3_ particles were subsequently labeled with fluorescent dextran (TRITC) by entrapping these molecules inside the porous CaCO_3_. To that end the CaCO_3_ microparticles were incubated in 0.5 mL of aqueous TRITC-dextran solution (1 mg/mL) for 30 min. Then particles were washed twice with water. In the final step two layers of the synthetic polyelectrolytes poly(allylamine hydrochloride) (PAH, 2 mg/mL in 0.5 M NaCl, positively charged) and poly(sodium 4-styrenesulfonate) (PSS, 2 mg/mL in 0.5 M NaCl, negatively charged) were adsorbed onto the surface of the CaCO_3_ particles via the layer-by-layer (LbL) technique [51–54]. Since the surface charge of cuboidal particles is almost neutral and in order to facilitate polyelectrolyte adsorption they were coated first with PSS to produce the following sequence of layers: (PSS/PAH/PSS). It should be pointed out that the CaCO_3_ cores were not dissolved at the end of the assembly procedure. Commercial, originally negatively charged silica particles (4.8 ± 0.2 μm, Microparticles GmbH, Germany) were coated with one layer of PAH, then incubated with TRITC-dextran and subsequently terminated with PSS. This time the fluorescent molecules labeled the shell of silica particles. Both CaCO_3_ and silica particles are terminated with a PSS shell and thus were negatively charged at neutral pH.

### Characterization of particles

The size of the particles was determined by optical microscopy, Fig. 1. The shape of dried particles was confirmed by taking scanning electron microscopy (SEM) and transmission electron microscopy (TEM) images, Fig. 1, the third and fourth row, respectively. The volumes (V) of differently shaped particles were calculated using the following formulas. For spherical particles ${\text{V}} = \frac{4}{3}\pi \left( {\frac{\text{d}}{2}} \right)^{3}$, where d = d_1_ = d_2_ is the particle diameter. For cuboidal particles ${\text{V}} = {\text{d}}_{ 1} \cdot {\text{d}}_{ 2}^{ 2}$, assuming “flat” particles as square base with side lengths d_1_ and a height of d_2_. For ellipsoidal particles ${\text{V}} = \frac{4}{3}\pi \frac{{{\text{d}}_{1} }}{2} \cdot \left( {\frac{{{\text{d}}_{2} }}{2}} \right)^{2}$ with the diameters d_1_ of the long axis and d_2_ of the short axis of an ellipsoid with prolate spheroid shape, respectively. All particles show symmetry around one axis. The extension of the particles along this axis is the particle height d_1_. The extensions of the particles perpendicular to this axis are the particle widths d_2_, *cf.* Fig. 1. The aspect ratio R was calculated as R = d_1_/d_2_, e.g. for “flat” particles R < 1, for spherical particles R = 1, and for elongated particles R > 1.

After the polyelectrolyte layer deposition (last PSS layer) the particles had neutral or negative zeta potential. For larger spherical particles the value was measured to be ~−41.4 ± 0.9 mV, while for small spheres, small ellipse and cubes the values were −23.4 ± 0.5, −28.2 ± 0.2 and −25.8 ± 0.6, respectively. Zeta-potentials for “big” elliptic particles were measured to be close to the neutral value.

### Cell experiments

For uptake studies cervical carcinoma HeLa cells 35,000 cells were seeded for 24 h on sterilized round cover slips (Carl Roth) placed on the bottom of 12-well plates with 18 mm diameter (TTP). Each well was filled with 700 µL of Eagle’s Minimum Essential Medium (EMEM), which contained 10% vol of fetal bovine serum (FBS), 1% vol of the antibiotics penicillin and streptomycin (P/S) at 37°C and 5% CO_2_ atmosphere. After the first 24 h, cells were incubated with differently shaped particles with a ratio of 10 particles/cell for another 24 h. The concentration of particles was calculated with a hemocytometer, which was possible due to the large size of the particles that allowed for visualization with optical microscopy. After that, cover slips were transferred to a flat surface covered with parafilm (Carl Roth) to proceed with the fixation of the cells and the staining of the cell compartments following several steps. Firstly, cells were fixed with 100 µL of a 4% paraformaldehyde solution for 30 min. Then, cells were permeabilized with 70 µL of a solution of 0.5 mg/mL saponin and 5 mg/mL glycine in phosphate buffered saline (PBS) for 5 min. After that, cells were blocked with additional 70 µL of 20 mg/mL solution of bovine serum albumin (BSA) for 30 min. After blocking the cells were incubated for 1 h with primary antibodies LAMP-1 (70 µL, 1 mg/mL) and washed three times with blocking solution. Then, cells were incubated for 1 h with (70 µL, 4 mg/mL) fluorophore-conjugated (DyLight 649) secondary antibodies, 4′-6-diamidino-2-phenylindole (DAPI), and phalloidin-tetramethylrhodamine. The secondary antibodies labeled with DyLight 649 were used to stain lysosomal compartments. DAPI was used to stain the nucleus and phalloidin to stain the cytoskeleton. In a last step, cells were washed three times with PBS and finally Fluoromount-G was used to fix the cover slips above cells on glass slides (76 × 26 mm) [24]. Fluorescence micrographs of HeLa cells with the internalized particles were recorded with a confocal microscope (Zeiss LSM 510 Meta Confocal Microscope) equipped with a laser diode emitting at 405 nm (used to visualize the nuclei labeled with DAPI, emission filter: LP 420), an argon laser with a line at 488 nm (used to visualize the cytoskeleton labeled with Oregon Green^®^ 488 phalloidin, emission filter BP 505–550) and a helium–neon laser for excitation at 543 nm (used to visualize the microparticles labeled with TRITC, emission filter: BP 560–615) and at 633 nm (used to visualize the lysosomes labeled with DyLight 649, emission filter LP 420), respectively. To reduce the scanning time we used the same channel and emission filter to visualize DAPI and DyLight 649. Lysosomes stained with DyLight 649 were artificially stained in yellow. Images were taken with a Plan-Apochromat 63×/1.40 Oil DIC M27 objective, and the pin hole was set to 0.86–1.32 airy units. All images were recorded and analyzed as z-stacks. The number of internalized particles per cell was then determined. In average, 600–700 particles were counted and at least 100 different cells were analyzed for every experiment that was repeated twice. Dividing and not fully depicted cells were not considered for analysis. Taking into account the staining of: (1) the actin network which delineated the cell shape (green fluorescence coming from fluorescently-labeled phalloidin), (2) the lysosomal compartments (artificial yellow color coming from DyLight 649-conjugated secondary antibody), and (3) the nucleus inside each cell (blue fluorescence coming from DAPI), microparticles (in red fluorescently-labeled) surrounded by DyLight 649 and phalloidin were classified as intracellular. Microparticles adherent to the outer cell plasma membrane and in between the grown cells were clearly distinguished from the microparticles with intracellular location, *cf.* Figs. 3 and 4 [32]. Based on these confocal images, histograms showing the number of internalized particles per cell f(N), as well as the corresponding cumulative distribution functions (CDFs) p(N) were generated, *cf.* Fig. 5 and Additional file 1: Figure S1. Standard deviations were calculated from the deviation of the independent f(N) or CDFs of two different experiments per each data set. The histogram f(N) describes the frequency f, i.e. the number of events, in which cells with exactly N internalized particles were observed. The CDF p(N) indicates the probability, that a cell contains N or less particles internalized. For example, p(N = 3) is the probability to find cells with either 0, 1, 2, or 3 internalized particles. CDFs are directly calculated from histograms where p(N) = $\mathop \sum \nolimits_{{{\text{i}} = 0}}^{\text{N}} {\text{f}}({\text{i}})$/$\mathop \sum \nolimits_{{{\text{i}} = 0}}^{\infty } {\text{f}}({\text{i}})$ and they are used to make the differences of particle uptake easier to be visualized. The CDFs are normalized and thus 0 ≤ p(N) ≤ 1 [30]. All data can be found in the Additional file 1.

## Additional file

Additional file 1:
**Figure S1.** Cumulative probability p(N) for the total internalized particle volume V_t_ per cell (p(V_t_)). Particles had different shape (and size): “small” ellipsoidal CaCO_3_ (black dots), “big” ellipsoidal CaCO_3_ (red dots), cuboidal CaCO_3_ (blue dots), “small” spherical CaCO_3_ (green dots) and “big” spherical SiO_2_ (pink dots). The plot is based on the data shown in Figure 9. The vertical bars represent the standard deviation values. **Figure S2.** Parameters of particles and cumulative probability. (a) Volume (V), surface (S) and surface-to-volume ratio (S/V) of the five different kinds of particles. (b) Cumulative probability plot p(S_t_) for total surface S_t_ of internalized particles per cell for particles with different shape (and size) after 24 h of particle incubation with a concentration of 10 particles added per cell. The total internal surface corresponds to the surface S of the particles times the number N of internalized particles per cell. Data are displayed for cuboidal CaCO_3_ (blue dots), “small” spherical CaCO_3_ (green dots), “big” spherical SiO_2_ (pink dots), “big” ellipsoidal CaCO_3_ (red dots), and “small” CaCO_3_ ellipsoidal particles (black dots). All particles had the same surface modification (positively charged layer of PAH). The vertical bars represent the standard deviation values. **Table SI-1.** Raw data of the histogram shown in Figure 5a for "small" and "big" ellipsoidal particles. f(N) is the frequency of cells which have internalized N particles per HeLa cell after 24 h of particle incubation with a concentration of 10 added particles per cell. SD stands for standard deviation. **Table SI-2.** Raw data of the cumulative probability plot shown in Figure 5b for "small" and "big" ellipsoidal particles. p(N) is the cumulative probability of cells, which have internalized N particles per HeLa cell after 24 h of particle incubation with a concentration of 10 added particles per cell. SD stands for standard deviation. **Table SI-3.** Raw data of the cumulative probability plot shown in Figure 5c for all types of particles. p(N) is the cumulative probability of cells, which have internalized N particles per HeLa cell after 24 h of particle incubation with a concentration of 10 added particles per cell. SD stands for standard deviation, sph. stands for spherical, ellip. stands for ellipsoidal and cub. stands for cuboidal. **Table SI-4.** Raw data of the cumulative total internalized particle volume V_t_ per cell (p(V_t_)) shown in Figure S1. SD stands for standard deviation, sph. stands for spherical, ellip. stands for ellipsoidal and cub. stands for cuboidal.

## Abbreviations

CaCO_3_: calcium carbonate
DAPI: diamidino-2-phenylindole
PSS: poly(sodium 4-styrenesulfonate) sodium salt
PAH: Poly(allylamine hydrochloride)
BSA: bovine serum albumin
EG: ethyleneglycol
