# Supplementary material for: The influence of the size and aspect ratio of anisotropic, porous CaCO3 particles on their uptake by cells
Source: J Nanobiotechnology. 2015 Sep 4;13:53. doi: 10.1186/s12951-015-0111-7 (PMC4558630; doi:10.1186/s12951-015-0111-7)
Supplement: Additional file 1: — Figure S1. Cumulative probability p(N) for the total internalized particle volume Vt per cell (p(Vt)). Particles had different shape (and size): “small” ellipsoidal CaCO3 (black dots), “big” ellipsoidal CaCO3 (red dots), cuboidal CaCO3 (blue dots), “small” spherical CaCO3 (green dots) and “big” spherical SiO2 (pink dots). The plot is based on the data shown in Figure 9. The vertical bars represent the standard deviation values. Figure S2. Parameters of particles and cumulative probability. (a) Volume (V), surface (S) and surface-to-volume ratio (S/V) of the five different kinds of particles. (b) Cumulative probability plot p(St) for total surface St of internalized particles per cell for particles with different shape (and size) after 24 h of particle incubation with a concentration of 10 particles added per cell. The total internal surface corresponds to the surface S of the particles times the number N of internalized particles per cell. Data are displayed for cuboidal CaCO3 (blue dots), “small” spherical CaCO3 (green dots), “big” spherical SiO2 (pink dots), “big” ellipsoidal CaCO3 (red dots), and “small” CaCO3 ellipsoidal particles (black dots). All particles had the same surface modification (positively charged layer of PAH). The vertical bars represent the standard deviation values. Table SI-1. Raw data of the histogram shown in Figure 5a for "small" and "big" ellipsoidal particles. f(N) is the frequency of cells which have internalized N particles per HeLa cell after 24 h of particle incubation with a concentration of 10 added particles per cell. SD stands for standard deviation. Table SI-2. Raw data of the cumulative probability plot shown in Figure 5b for "small" and "big" ellipsoidal particles. p(N) is the cumulative probability of cells, which have internalized N particles per HeLa cell after 24 h of particle incubation with a concentration of 10 added particles per cell. SD stands for standard deviation. Table SI-3. Raw data of the cumulative probability [file 12951_2015_111_MOESM1_ESM.docx]

Supporting Information

**The Influence of the Size and Aspect Ratio of Anisotropic, Porous CaCO_3_ Particles on their Uptake by Cells**

Bogdan Parakhonskiy^1,2¶^, Mikhail V. Zyuzin^3¶^, Alexey Yashchenok^2,4^, Susana Carregal-Romero^3^, Joanna Rejman^3^, Helmuth Möhwald^4^, Wolfgang J. Parak^3^*, Andre G. Skirtach^4,5,6^*

^1^ Shubnikov Institute of Crystallography, Russian Academy of Science, Moscow, Russia

^2^ Institute of Nanostructures and Biosystems, Saratov State University, Saratov, Russia

^3^ Fachbereich Physik, Philipps University of Marburg, Marburg, Germany

^4^ Department of Interfaces, Max-Planck Institute of Colloids and Interfaces, Potsdam, Germany

^5^ NanoBio-Photonics, Ghent University, Ghent, Belgium

^6^ Department of Molecular Biotechnology, Ghent University, Ghent, Belgium

¶ These authors contributed equally to this work.

**Figure S1.** **Cumulative propability p(N) for the total internalized particle volume V_t_ per cell (p(V_t_)).** Particles had different shape (and size): “small” ellipsoidal CaCO_3_ (black dots), “big” ellipsoidal CaCO_3_ (red dots), cuboidal CaCO_3_ (blue dots), “small” spherical CaCO_3_ (green dots) and “big” spherical SiO_2_ (pink dots). The plot is based on the data shown in Figure 9. The vertical bars represent the standard deviation values.

**Figure S2.** **Parameters of particles and cumulative probability**. (a) Volume (V), surface (S) and surface-to-volume ratio (S/V) of the five different kinds of particles. (b) Cumulative probability plot p(St) for total surface S_t_ of internalized particles per cell for particles with different shape (and size) after 24 h of particle incubation with a concentration of 10 particles added per cell. The total internal surface corresponds to the surface S of the particles times the number N of internalized particles per cell. Data are displayed for cuboidal CaCO_3_ (blue dots), “small” spherical CaCO_3_ (green dots), “big” spherical SiO_2_ (pink dots), “big” ellipsoidal CaCO_3_ (red dots), and “small” CaCO_3_ ellipsoidal particles (black dots). All particles had the same surface modification (positively charged layer of PAH). The vertical bars represent the standard deviation values.

| **N** | **f (N),**  **small ellipsoidal** | **SD** | **f (N),**  **big ellipsoidal** | **SD** |
| --- | --- | --- | --- | --- |
| 0 | 0.39 | 0.54 | 8.6 | 1.80 |
| 1 | 2.74 | 0.51 | 10.69 | 6.00 |
| 2 | 5.10 | 0.64 | 11.37 | 4.71 |
| 3 | 7.83 | 2.10 | 12.75 | 7.83 |
| 4 | 8.20 | 3.74 | 14.13 | 8.20 |
| 5 | 9.00 | 2.62 | 12.06 | 9.00 |
| 6 | 10.58 | 0.38 | 8.62 | 5.07 |
| 7 | 10.23 | 4.61 | 4.80 | 3.44 |
| 8 | 8.65 | 2.36 | 3.44 | 2.29 |
| 9 | 7.46 | 0.68 | 2.41 | 3.44 |
| 10 | 5.89 | 0.65 | 2.75 | 2.07 |
| 11 | 5.10 | 0.645 | 1.72 | 0.96 |
| 12 | 4.71 | 0.08 | 1.37 | 4.71 |
| 13 | 4.32 | 0.63 | 1.72 | 4.32 |
| 14 | 4.71 | 0.08 | 1.72 | 2.87 |
| 15 | 5.09 | 0.47 | 1.72 | 3.74 |

**Table SI-1.** **Raw data of the histogram shown in Figure 5a for "small" and "big" ellipsoidal particles.** f(N) is the frequency of cells which have internalized N particles per HeLa cell after 24 h of particle incubation with a concentration of 10 added particles per cell. SD stands for standard deviation.

| **N** | **p(N),**  **small ellipsoidal** | **SD** | **p(N),**  **big ellipsoidal** | **SD** |
| --- | --- | --- | --- | --- |
| 0 | 0.00 | 0.00 | 0.09 | 0.04 |
| 1 | 0.03 | 0.01 | 0.19 | 0.07 |
| 2 | 0.08 | 0.04 | 0.31 | 0.03 |
| 3 | 0.16 | 0.04 | 0.43 | 0.03 |
| 4 | 0.24 | 0.06 | 0.58 | 0.04 |
| 5 | 0.33 | 0.09 | 0.70 | 0.06 |
| 6 | 0.44 | 0.09 | 0.78 | 0.06 |
| 7 | 0.54 | 0.05 | 0.83 | 0.03 |
| 8 | 0.63 | 0.02 | 0.86 | 0.02 |
| 9 | 0.70 | 0.02 | 0.90 | 0.02 |
| 10 | 0.76 | 0.01 | 0.92 | 0.03 |
| 11 | 0.81 | 0.01 | 0.93 | 0.02 |
| 12 | 0.86 | 0.01 | 0.95 | 0.02 |
| 13 | 0.90 | 0.01 | 0.96 | 0.01 |
| 14 | 0.95 | 0.01 | 0.98 | 0.01 |
| 15 | 1 | 0 | 1 | 0 |

**Table SI-2.** **Raw data of the cumulative probability plot shown in Figure 5b for "small" and "big" ellipsoidal particles.** p(N) is the cumulative probability of cells, which have internalized N particles per HeLa cell after 24 h of particle incubation with a concentration of 10 added particles per cell. SD stands for standard deviation.

| **N** | **p(N),**  **small sph.** | **SD** | **p(N),**  **small ellip.** | **SD** | **p(N), cub.** | **SD** | **p(N),**  **big ellip.** | **SD** | **p(N),**  **big sph.** | **SD** |
| --- | --- | --- | --- | --- | --- | --- | --- | --- | --- | --- |
| 0 | 0.12 | 0.02 | 0.004 | 0.005 | 0.24 | 0.05 | 0.09 | 0.04 | 0.09 | 0.03 |
| 1 | 0.25 | 0.08 | 0.03 | 0.01 | 0.51 | 0.12 | 0.19 | 0.07 | 0.23 | 0.01 |
| 2 | 0.39 | 0.12 | 0.08 | 0.04 | 0.67 | 0.10 | 0.31 | 0.03 | 0.412 | 0.004 |
| 3 | 0.52 | 0.11 | 0.16 | 0.04 | 0.78 | 0.04 | 0.43 | 0.03 | 0.511 | 0.005 |
| 4 | 0.63 | 0.10 | 0.24 | 0.06 | 0.86 | 0.01 | 0.58 | 0.04 | 0.58 | 0.03 |
| 5 | 0.70 | 0.09 | 0.33 | 0.09 | 0.92 | 0.01 | 0.70 | 0.06 | 0.66 | 0.02 |
| 6 | 0.75 | 0.09 | 0.44 | 0.09 | 0.95 | 0.01 | 0.78 | 0.06 | 0.71 | 0.02 |
| 7 | 0.82 | 0.07 | 0.54 | 0.05 | 0.97 | 0.01 | 0.83 | 0.03 | 0.76 | 0.01 |
| 8 | 0.86 | 0.06 | 0.63 | 0.02 | 0.99 | 0.01 | 0.86 | 0.02 | 0.817 | 0.003 |
| 9 | 0.90 | 0.04 | 0.70 | 0.02 | 1 | 0 | 0.89 | 0.02 | 0.84 | 0.02 |
| 10 | 0.92 | 0.03 | 0.76 | 0.01 | 1 | 0 | 0.92 | 0.03 | 0.87 | 0.03 |
| 11 | 0.95 | 0.03 | 0.81 | 0.01 | 1 | 0 | 0.93 | 0.02 | 0.90 | 0.03 |
| 12 | 0.96 | 0.02 | 0.86 | 0.01 | 1 | 0 | 0.95 | 0.02 | 0.91 | 0.03 |
| 13 | 0.97 | 0.01 | 0.90 | 0.01 | 1 | 0 | 0.96 | 0.01 | 0.93 | 0.03 |
| 14 | 0.98 | 0.01 | 0.95 | 0 | 1 | 0 | 0.98 | 0.01 | 0.96 | 0.013 |
| 15 | 1 | 0 | 1 | 0 | 1 | 0 | 1 | 0 | 1 | 0 |

**Table SI-3.** **Raw data of the cumulative probability plot shown in Figure 5c for all types of particles.** p(N) is the cumulative probability of cells, which have internalized N particles per HeLa cell after 24 h of particle incubation with a concentration of 10 added particles per cell. SD stands for standard deviation, sph. stands for spherical, ellip. stands for ellipsoidal and cub. stands for cuboidal.

| **V_t_,**  **µm^3^** | **p(N),**  **small sph.** | **SD** | **V_t_, µm^3^** | **p(N), small ellip.** | **SD** | **V_t_,**  **µm^3^** | **p(N), cub.** | **SD** | **V_t_, µm^3^** | **p(N),**  **big ellip.** | **SD** | **V_t_, µm^3^** | **p(N),**  **big sph.** | **SD** |
| --- | --- | --- | --- | --- | --- | --- | --- | --- | --- | --- | --- | --- | --- | --- |
| 0 | 0.12 | 0.02 | 0 | 0.004 | 0.005 | 0 | 0.24 | 0.05 | 0 | 0.09 | 0.04 | 0 | 0.09 | 0.03 |
| 18.8 | 0.25 | 0.08 | 0.7 | 0.03 | 0.01 | 25.6 | 0.5 | 0.1 | 4.7 | 0.19 | 0.07 | 57.9 | 0.23 | 0.01 |
| 37.6 | 0.4 | 0.1 | 1.4 | 0.08 | 0.04 | 51.2 | 0.7 | 0.1 | 9.4 | 0.31 | 0.03 | 115.8 | 0.412 | 0.004 |
| 56.4 | 0.5 | 0.1 | 2.1 | 0.16 | 0.04 | 76.8 | 0.78 | 0.04 | 14.1 | 0.43 | 0.03 | 173.7 | 0.511 | 0.005 |
| 75.2 | 0.62 | 0.09 | 2.8 | 0.24 | 0.06 | 102.4 | 0.860 | 0.002 | 18.8 | 0.58 | 0.04 | 231.6 | 0.58 | 0.03 |
| 94 | 0.70 | 0.09 | 3.5 | 0.33 | 0.09 | 128 | 0.92 | 0.01 | 23.5 | 0.70 | 0.06 | 289.5 | 0.66 | 0.027 |
| 112.8 | 0.75 | 0.09 | 4.2 | 0.44 | 0.09 | 153.6 | 0.95 | 0.01 | 28.2 | 0.78 | 0.06 | 347.4 | 0.71 | 0.02 |
| 131.6 | 0.82 | 0.07 | 4.9 | 0.54 | 0.05 | 179.2 | 0.973 | 0.007 | 32.9 | 0.83 | 0.03 | 405.3 | 0.76 | 0.01 |
| 150.4 | 0.86 | 0.06 | 5.6 | 0.63 | 0.02 | 204.8 | 0.995 | 0.007 | 37.6 | 0.86 | 0.02 | 463.2 | 0.82 | 0.00259 |
| 169.2 | 0.90 | 0.04 | 6.3 | 0.70 | 0.02 | 230.4 | 1 | 0 | 42.3 | 0.89 | 0.02 | 521.1 | 0.84 | 0.02 |
| 188 | 0.92 | 0.03 | 7 | 0.76 | 0.01 | 256 | 1 | 0 | 47.0 | 0.92 | 0.03 | 579 | 0.874 | 0.03 |
| 206.8 | 0.95 | 0.03 | 7.7 | 0.81 | 0.01 | 281.6 | 1 | 0 | 51.7 | 0.93 | 0.02 | 636.9 | 0.90 | 0.03 |
| 225.6 | 0.96 | 0.02 | 8.4 | 0.86 | 0.01 | 307.2 | 1 | 0 | 56.4 | 0.95 | 0.02 | 694.8 | 0.91 | 0.03 |
| 244.4 | 0.97 | 0.01 | 9.1 | 0.90 | 0.01 | 332.8 | 1 | 0 | 61.1 | 0.96 | 0.01 | 752.7 | 0.93 | 0.03 |
| 263.2 | 0.98 | 0.01 | 9.8 | 0.949 | 0.005 | 358.4 | 1 | 0 | 65.8 | 0.98 | 0.01 | 810.6 | 0.96 | 0.01 |
| 282 | 1 | 0 | 10.5 | 1 | 0 | 384 | 1 | 0 | 70.5 | 1 | 0 | 868.5 | 1 | 0 |

**Table SI-4.** **Raw data of the cumulative total internalized particle volume V_t_ per cell (p(V_t_)) shown in Figure S1.** SD stands for standard deviation, sph. stands for spherical, ellip. stands for ellipsoidal and cub. stands for cuboidal.
